# SARS-CoV-2 and monkeypox: what is common and what is not in a present pandemic versus a potential one—a neuropsychiatric narrative review

**DOI:** 10.1186/s41983-022-00563-w

**Published:** 2022-11-08

**Authors:** Tamer Roushdy

**Affiliations:** Neurology Department, Faculty of Medicine, Ain Shams University, 38 Abbasia, Cairo, 11591 Egypt

**Keywords:** SARS-CoV-2 virus, Coronavirus, Pandemic, Monkeypox virus, Neuropsychiatric manifestations, Virology, Mortality and morbidity, Quarantine, Stigma

## Abstract

Pandemic represents challenging medical emergency as it is usually associated with high rates of mortalities and morbidities. Along the last 2 and half years the world has faced the emergence of severe acute respiratory syndrome corona virus 2 pandemic that caught medical agencies and health authorities by surprise and costed more than half billion morbidities and 6 million mortalities. Unfortunately, the way developed countries contained the novel corona virus was unsatisfactory in means of early quarantines as well as obtaining and distributing an effective vaccine. This failure in management might have been responsible for the emergence of a new potential pandemic caused by monkeypox virus. Along the current review article, a detailed comparison is presented between corona virus and monkeypox virus based on virological characteristics, role of corona virus in monkeypox spread, pathogenesis, neuropsychiatric manifestations, and treatment and management. It is obvious that both viruses have a major role in causing various neuropsychiatric manifestations. Neurological manifestations are either bound directly to the virus spread to central and peripheral nervous system or secondary to triggering an immune reaction. Psychiatric ones are mostly related to stigmatization, isolation as well as changes that takes place in neurotransmitters and their metabolites within the nervous system. Dealing properly with monkeypox virus spread through previously learned lessons from corona virus might protect the world from a new pandemic.

## Introduction

Pandemic is an infectious disease that originates in a confined area (single country or region) under the term epidemic state then spreads on a wide scale to involve different countries, regions, and even continents affecting a large proportion of world’s population [1].

Meanwhile an endemic is a prevalent infectious disease within a specific population confined to geographical area [2].

An example of an endemic disease is malaria that is present mainly in sub-Saharan Africa besides broad geographical zones along the equator yet nearly absent in the rest of the world due to absence of its transmitting vector [3].

Unlike malaria, cholera is another infectious disease that is endemic in sub-Saharan Africa with an outbreak epidemic that can turn to become pandemic if not properly managed. Historically cholera had seven pandemic attacks the latest was in 1961 with emergence of a new strain [4].

Epidemics usually occur secondary to different reasons as reduced general hygiene or low immune system in a community. An example of an epidemic is the cholera epidemic outbreak of Yemen that occurred as a result of the Yemeni war [5].

So, it could be concluded that pandemics originate from uncontrolled epidemics. Since the year 2020 the world is facing a pandemic that originated from a central market in Wuhan city in China that sells food besides animals and other products. It is caused by a novel corona virus and was termed after its commonest presentation being severe acute respiratory syndrome of corona virus 2 (SARS-CoV-2) [6].

SARS-CoV-2 belongs to the same corona viruses that emerged in the beginning of the twenty first century which were termed severe acute respiratory syndrome of corona virus (SARS-CoV) and middle east respiratory syndrome of corona virus (MERS-CoV). SARS-CoV-2 is highly infectious if compared to its earlier ancestors since its lipid envelope where the protein spikes are emerging has some changes in its fusion sites that facilitate better attachment to cells and augment entrance [7].

Moreover, it is continuously mutating and this further facilitates its ability to escape acquired immunity from previous attacks within the same individual and being a novel virus to humans there is already no innate immunity against it.

For such reasons, SARS-CoV-2 pandemic is still running for more than 2 years affecting different body systems with a wide range of autoimmune conditions and diverse neuropsychiatric manifestations that are either a result of direct viral effect or secondary to excessive inflammatory response or crossed immunological reaction [8].

Meanwhile the world is facing another unfamiliar outbreak of a previously considered endemic which is monkeypox (MPX) that has crossed borders in a behavior that is uncommon for endemic diseases [9, 10].

According to World Health Organization (WHO), monkeypox is now considered a health emergency of global concern. MPX also can cause some neuropsychiatric manifestations. Although such manifestations are not fully understood. Yet, it shares similar general viremia related symptoms as well as psychiatric stigmatization that is originating out of fear from an unknown pathogen, its effect and to what extent it is contagious [11–13].

Along the current review a comparative presentation between SARS-CoV-2 and MPX regarding their cross-relation, features and neuropsychiatric manifestations as well as ways of management and control is presented (Table 1).

**Table 1:** Similarities and differences between SARS-CoV-2 and MPX

	SARS-CoV-2	MPX
Virological classification	Orthocoronavirinae subfamily, Coronaviridae family	Chordopoxvirinae subfamily, Poxviridae family
Nucleic acid structure	Single stranded RNA	Double stranded DNA
Forms	Alpha, Beta, Gamma, Delta, Omicron with its different clades	Nigerian clade and democratic republic of Congo clade
Abundant form worldwide	Omicron	Nigerian clade
Way of transmission	Droplet, direct contact, aerosol	Long-term close direct contact with infected animals, contact with infected body fluids, human-to-human transmission among homosexual men
Nature of spread	Tides and waves	Constant rise
Source of body entrance	Attaching its surface spikes to ACE2 receptors on endothelial cells	Macropinocytosis
Commonest neuropsychiatric manifestations	Strokes, encephalitis, peripheral neuropathy, autoimmune neuropathy, fatigue, lack of concentration, inattention, memory lapses, generalized weakness, sleep disturbance, depression, anxiety, post-traumatic stress, chronic headache	Headache, myalgia, photophobia, pain and fatigue, seizures, encephalitis, anxiety, depression up to suicide
Manifestations in pediatrics	Less aggressive yet rarely MIS-C can develop	More aggressive

SARS-CoV-2: severe acute respiratory syndrome corona virus 2, MPX: monkeypox virus, RNA: ribonucleic acid, DNA: deoxyribonucleic acid, ACE2: angiotensin converting enzyme 2 receptors, MIS-C: multisystem inflammatory syndrome in children

## Main text

### SARS-CoV-2 and MPX virology

SARS-CoV-2 belongs to the orthocoronavirinae subfamily that is derived from the Coronaviridae family. It is a single stranded RNA virus that is enveloped in a bilayer of lipid structured envelope with spikes on its surface [14].

SARS-CoV-2 is considered one of the largest known RNA viruses reaching 29.9 kb. This makes it difficult to be transmitted through air-borne transmission, yet it could be transmitted through droplet transmission as well as direct contact with infected surfaces or through aerosol transmission [15].

RNA viruses are liable to mutation secondary to errors that develop in their genetic codes while replicating. Such mutation can result in new strains making it difficult to control a pandemic caused by RNA viruses [16]. SARS-CoV-2 has mutated from its original alpha form to beta, gamma, delta, and omicron with its different clades within the last 2 years [17].

Mutations in SARS-CoV-2 are associated with rapid spread and such spread up till now has infected more than half billion mankind with more than 6 million deaths.

The way through which SARS-CoV-2 gain entrance to the human body is through attaching its spikes to the endothelial cells angiotensin converting enzyme 2 receptors (ACE-2) that are abundant along different organs beginning from the nasal cells, respiratory system, renal system, cardiac, vascular system and nervous system [6].

As for MPX, it belongs to the chordopoxvirinae subfamily that is derived from the Poxviridae family. Unlike SARS-CoV-2, monkeypox is a double stranded 197 kb DNA virus with a lipoprotein envelope and it enters cells through macropinocytosis. Being a DNA virus makes it stable when compared to SARS-CoV-2 as DNA viruses have the ability to proof-read their genome while replicating with a less chance to have mutated forms although not fully impossible [18].

Along its original endemic site being west and central Africa, MPX virus has two clades, one in Nigeria with less mortality rates being 1/100 cases and the other in Democratic Republic of Congo with mortality reaching 1/10 cases while Cameron act as a border zone with the two clades endemic in it [18].

Along its current 2022 cross-border outbreak, MPX had a 15 single nucleotide polymorphism mutation which is an uncommon and a relatively rapid mutation for a DNA virus [19].

**Fig. 1:**
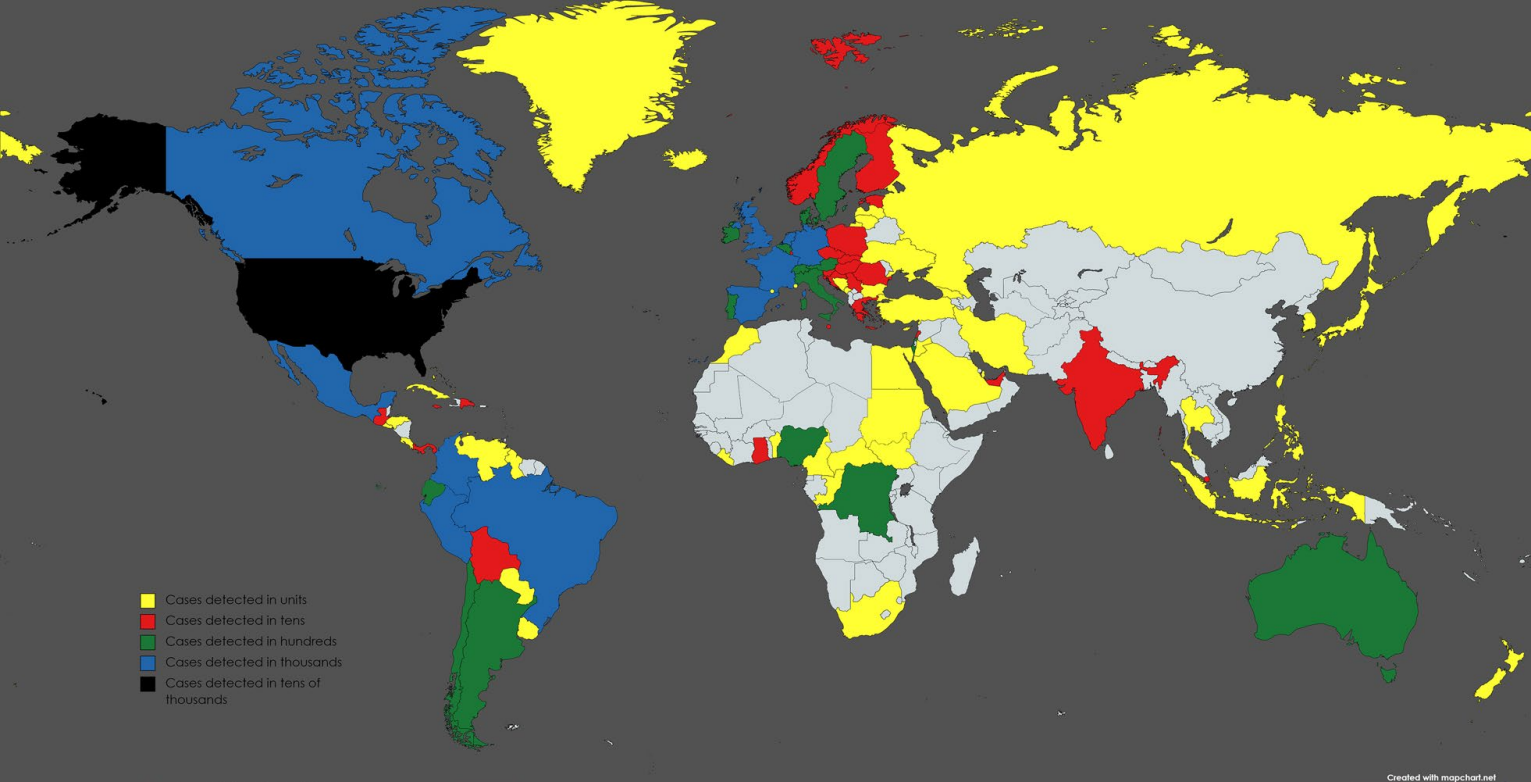
World map showing countries with monkeypox cases based on numbers that is obtained from centers of disease control and prevention by 28th of September 2022. countries in yellow represent that numbers of cases detected in single digit (units), red represent detected cases in tens, green represent detected cases in hundreds, blue represent detected cases in thousands, and black represent detected cases in tens of thousands

Monkeypox spread through close long-term contact with an infected animal as well as through body fluids, until the current 2022 global outbreak with a total of more than 60,000 cases worldwide with only few hundred cases in endemic countries there was no direct wide spread human-to-human transmission [20, 21] (Fig. 1, Table 2). Whether such direct spread among humans who have never traveled to the endemic areas along Nigeria where the running clade worldwide is present is related to SARS-CoV-2 is a question of concern.

**Table 2:** Countries with reported cases of spreading monkeypox based on centers for disease control and prevention from 17th of August to 28th of September 2022

	11th Aug	16th Aug	22nd Aug	27th Aug	6th Sept	10th Sept	15th Sept	20th Sept	28th Sept
Countries/total	31,803	36,591	41,360	44,507	53,031	56,613	56,935	62,411	66,554
Andorra	4	4	4	4	4	4	4	4	4
Argentina	37	49	72	72	170	170	170	265	326
Aruba	0	0	0	0	2	2	2	3	3
Australia	58	70	89	89	125	125	125	132	135
Austria	175	200	217	231	271	278	278	300	309
Bahamas	1	1	1	2	2	2	2	2	2
Barbados	1	1	1	1	1	1	1	1	1
Belgium	546	546	624	671	706	726	726	744	757
Benin	3	3	3	3	3	3	3	3	3
Bermuda	1	1	1	1	1	1	1	1	1
Bolivia	5	11	31	42	79	89	89	129	175
Bosnia and Herzegovina	1	3	3	3	3	3	3	3	5
Brazil	2131	2584	3359	3788	5037	5525	5525	6649	7445
Bulgaria	4	4	4	4	4	5	5	6	6
Cameroon	7	7	7	7	7	7	7	7	8
Canada	957	1059	1112	1173	1251	1317	1317	1363	1389
Central African Republic	8	8	8	8	8	8	8	8	8
Chile	91	141	189	207	381	450	450	728	842
Colombia	55	84	129	273	582	938	938	1260	1653
Costa Rica	3	3	3	3	3	3	3	4	4
Croatia	12	17	17	22	26	27	27	27	29
Curaçao	0	0	1	1	1	1	1	1	3
Cuba	0	0	0	1	2	2	2	2	3
Cyprus	3	3	4	4	5	5	5	5	5
Czechia	29	35	36	41	48	58	58	62	66
Democratic Republic of Congo	163	163	163	163	195	195	195	195	195
Denmark	126	151	163	169	175	181	181	183	184
Dominican Republic	4	5	6	7	7	7	7	31	31
Ecuador	10	17	19	35	53	53	53	68	120
Egypt	0	0	0	0	0	1	1	1	1
El Salvador	0	0	0	0	1	1	1	2	5
Estonia	9	9	9	10	10	10	10	11	11
Finland	22	22	22	22	22	30	30	33	33
France	2423	2673	2889	2889	3558	3646	3646	3898	3970
Georgia	1	1	2	2	2	2	2	2	2
Germany	2982	3142	3266	3329	3493	3518	3519	3563	3607
Ghana	35	35	47	47	76	76	76	84	84
Gibraltar	5	6	6	6	6	6	6	6	6
Greece	41	49	50	52	58	66	66	69	74
Greenland	0	2	2	2	2	2	2	2	2
Guadeloupe	1	1	1	1	1	1	1	1	1
Guatemala	2	3	3	4	8	11	11	12	20
Guyana	0	0	0	0	2	2	2	2	2
Honduras	0	3	3	3	4	4	4	4	6
Hong Kong	0	0	0	0	0	1	1	1	1
Hungary	51	57	62	64	70	71	71	75	77
Iceland	11	11	12	12	12	12	12	12	14
India	9	9	9	9	10	10	10	12	12
Indonesia	0	0	0	1	1	1	1	1	1
Iran	0	0	1	1	1	1	1	1	1
Ireland	101	101	113	128	144	160	160	173	178
Israel	166	189	194	213	239	241	241	247	250
Italy	599	644	689	714	760	787	787	828	846
Jamaica	3	4	4	4	5	9	9	12	14
Japan	3	4	4	4	4	4	4	4	4
Jordan	3	4	4	4	4	4	4	4	4
Latvia	3	3	4	4	4	4	4	4	5
Lebanon	6	6	6	6	6	8	8	11	11
Liberia	2	2	2	2	2	2	2	3	3
Lithuania	3	5	5	5	5	5	5	5	5
Luxembourg	34	43	45	47	53	53	53	55	55
Malta	30	30	31	31	31	33	33	33	33
Martinique	1	1	1	1	1	1	1	1	1
Mexico	91	147	252	252	504	788	804	1050	1367
Moldova	1	1	2	2	2	2	2	2	2
Monaco	0	1	1	3	3	3	3	3	3
Montenegro	1	1	1	2	2	2	2	2	2
Morocco	1	1	1	1	3	3	3	3	3
Netherlands	959	1025	1087	1087	1166	1195	1199	1209	1221
New Caledonia	1	1	1	1	1	1	1	1	1
New Zealand	3	4	4	4	4	5	5	5	9
Nigeria	157	157	157	157	220	220	220	277	277
Norway	66	72	76	76	82	82	82	89	92
Panama	2	2	4	7	10	12	12	12	14
Paraguay	0	0	0	0	1	1	1	1	1
Peru	547	775	937	1188	1531	1724	1724	2054	2423
Philippines	1	1	1	4	4	4	4	4	4
Poland	85	95	104	121	130	145	145	160	182
Portugal	710	770	810	810	871	871	871	898	917
Qatar	3	3	3	3	3	3	3	4	5
Republic of Congo	3	3	3	3	3	3	3	5	5
Romania	28	32	33	35	36	36	36	37	40
Russia	1	1	1	1	1	1	1	2	2
Saint Martin	1	1	1	1	1	1	1	1	1
Saudi Arabia	5	5	6	6	8	8	8	8	8
Serbia	23	23	23	31	31	31	31	31	40
Singapore	15	15	15	15	16	16	16	19	19
Slovakia	8	10	10	12	12	14	14	14	14
Slovenia	40	43	43	43	43	45	45	46	47
South Africa	3	3	4	4	5	5	5	5	5
South Korea	1	1	1	1	1	2	2	2	2
South Sudan	0	0	0	0	0	2	2	2	2
Spain	5162	5719	5792	6284	6543	6749	6749	6947	7122
Sudan	1	1	1	2	2	2	2	6	7
Sweden	123	129	139	150	161	165	165	179	186
Switzerland	347	387	392	416	476	480	480	494	513
Taiwan	3	3	3	3	3	3	3	3	3
Thailand	4	4	5	5	7	7	7	8	8
Turkey	1	1	1	1	1	1	1	1	1
Ukraine	0	0	0	0	0	0	0	1	3
United Arab Emirates	16	16	16	16	16	16	16	16	16
United Kingdom	2914	3017	3081	3207	3413	3484	3484	3552	3585
United States	9492	11,889	14,594	15,908	19,961	21,504	21,805	23,892	25,340
Uruguay	2	2	2	2	4	5	5	5	6
Venezuela	1	1	1	1	3	3	3	3	5

### Is MPX spread related to SARS-CoV-2?

Infection with SARS-CoV-2 is associated with reduction in total leucocytic count and lymphopenia in many cases. Such low circulatory immune cells count can make infected cases with SARS-CoV-2 more liable for co-infection with MPX and this in turn may be associated with change in form and course of infection in one or both diseases as well as response to vaccination [22].

Another link between SARS-CoV-2 and MPX outbreak is absence of immunity in young generations against smallpox virus since stopping its vaccination campaigns after its eradication in 1980. Smallpox belongs to the same family of monkeypox and stopping the administration of its vaccine aided in relative increase in monkeypox cases in its endemic areas together with reduction in immunity of local residents on top of spreading SARS-CoV-2 that is associated as well in increasing numbers of monkeypox cases [23].

The world failure to equally distribute vaccines against SARS-CoV-2 among developing and developed countries plays a role in increasing the liability for catching SARS-CoV-2 that further reduces the patient’s immunity and increase his liability to have a co-infection with monkeypox. This failure in vaccine distribution is causing backfire on developed world with the cross-border spread of an increasing number of MPX cases [23, 24].

Another possible explanation for the outbreak of endemic Nigerian clade of MPX through many countries worldwide with most of them within the western world is the reduction in restrictions related to travel and lockdown after reaching more than 70% vaccination against SARS-CoV-2 in the developed world and the mass gathering that reached up to 80,000 attendants most of them from the gay and bisexual community along Maspalomas festival in the Canary Island between 5 and 15th May 2022. Such gathering may have played a role in the spread of viruses like MPX in case of the presence of one or more carrier within such gathering [23].

### Pathogenesis of SARS-CoV-2 and MPX neuropsychiatric manifestations

**Fig. 2:**
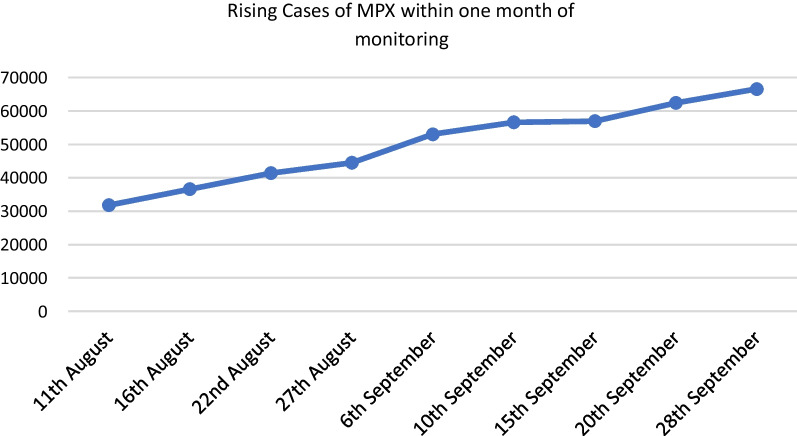
Rising cases of monkeypox within 1 month of monitoring

SARS-CoV-2 pandemic that is in the form of tides and waves which differs from MPX more linear spread (Fig. 2) have aided different health authorities and organizations to study its pathogenesis along different body systems with nervous system one of them.

Besides gaining entrance to cells through ACE-2 receptors which causes direct invasion, the commonest pathophysiologic mechanisms responsible for central and to an extent peripheral neurological manifestations of SARS-CoV-2 are attributed to exposure of the immune system to a novel virus with absence of innate immunity previous experience, which is followed by over activity and overexpression of cytokine and chemokine responses that reaches up to triggering autoimmune reactions [25].

Such overexpression of inflammatory and immune markers is reported along different studies that proved the presence of elevated values of interleukin-8 (IL-8) and tumor necrosis factor-alpha (TNF-α) besides pleocytosis in examined cerebrospinal fluid (CSF) of SARS-CoV-2 victims [26].

Central nervous system invasion of SARS-CoV-2 is through two routes, first is through hematological spread, where the virus reaches the endothelial cells lining the blood capillaries of the blood brain barrier (BBB) and invades such cells through attaching its spikes to ACE-2 receptors that are abundant within the endothelial cell surfaces and this causes break down in the BBB which in turns causes direct inflammation to the brain and brain edema that could compress vital centers along the brainstem with further affection of respiratory and circulatory systems. Second route is retrospectively either from the endothelial cells lining the olfactory bulb and along the olfactory pathway to the CNS or from chemo and mechanoreceptors of the respiratory system to the brainstem cardiorespiratory system [27–29].

Pathogenesis behind peripheral nervous system disorders associated with SARS-CoV-2 is either secondary to direct viral infection and that is why some literature reported cases were having a para-infectious presentation or secondary to triggering the immune system through molecular mimicry to attack the myelin sheath with post-infectious presentation [30–32].

Psychiatric manifestations presenting with SARS-CoV-2 is attributed to the lockdown and curfew that was declared along the initial waves of the pandemic which deprived medically chronic patients of their usual checkups with their treating physicians besides the media effect of presenting mortalities with daily counts that had direct mental traumatizing effect. Social distancing is also a main trigger to frustration, aggression, mood disorders, insomnia and psychosis [33, 34].

Stigma from being infected with fear of transmitting infection also played a role in overexpression of psychiatric manifestations besides the changes that develop along neurotransmission pathway and its metabolites triggered indirectly by stress and directly by the immune response effect to the virus on such neurotransmitters [35].

Kucukkarapinar and colleagues succeeded in linking psychiatric manifestations that takes place post SARS-CoV-2 exposure to the kynurenine pathway and tryptophan metabolism. Infection with SARS-CoV-2 alter the tryptophan pathway with increase in kynurenic acid, kynurenine, and quinolinic acid and decrease in tryptophan which in turn increases oxidative stress, and impairs glutamate action with a net affection on cognitive functions. Meanwhile increase in conversion of tryptophan to kynurenine is associated with reduction in serotonin that can explains psychiatric manifestations accompanying infection with SARS-CoV-2 as depression and anxiety [35].

As for neurological manifestations pathogeneses of MPX up till now are mainly attributed to inflammatory viremia effects of the virus causing fever that in turns affect muscles leading to myalgia. Raised body temperature is associated with tachycardia, tachypnea, hypercapnia up to hypoxia which affects all body systems and can cause headache, encephalopathy up to seizures in vulnerable cases [36].

Psychiatric pathogeneses of MPX are to a great extent stigma related especially that most available data are speaking on homosexuality role in transmission of MPX. And since it is a relatively recently human-to-human transmitted disease, its stigma originates from lacking full medical knowledge about it that resembles what epilepsy had in the ancient civilization thoughts [37].

### Neuropsychiatric aspects of SARS-CoV-2 and MPX

Unlike its name, SARS-CoV-2 does not just cause influenza like manifestations. Along the last 2 and half years since its declaration as a pandemic by WHO in March 2020, SARS-CoV-2 affected the nervous system in a plenty of ways.

Such affection is either central or peripheral, sometimes preceded the respiratory manifestations, and in other occasions is either conjoint with it or appears few weeks following diagnosis [8].

These different phases of neurological affection spotlight on the diverse pathophysiology of neurological complications of SARS-CoV-2 [8, 38].

Neurovascular complications of SARS-CoV-2 whether in the form of ischemic strokes or hemorrhagic ones and whether arterial or venous are very common and are reported with mild, moderate, or severe forms of SARS-CoV-2 [39, 40].

Stroke in some cases is the only manifestation of SARS-CoV-2. Its pathophysiology was described by Roushdy and Hamid which is related to uncontrolled rise in blood pressure secondary to down regulation in ACE-2 receptors that are used by SARS-CoV-2 spikes to gain entrance to endothelial cells, reduction in platelet count, disturbance in coagulation parameters whether procoagulants or anticoagulants as well as cytokine storm and elevated inflammatory biomarkers that can affect vessel wall integrity and induce vasculitis [6].

Besides the usual symptoms accompanying any viremia being myalgia, bony aches, fatigue, anorexia and fever, it is noticed that SARS-CoV-2 is associated with anosmia in many cases. Anosmia is believed to be secondary to viral invasion of olfactory nerve endings within the cribriform plate that is lined by endothelium cells abundant with ACE-2 [41].

Besides dysfunction of the olfactory nerve, other cranial nerves are occasionally affected following SARS-CoV-2 infection and secondary to long-term treatment with steroids in patients who are usually immunocompromised. Such patients might catch rhino-orbito-cerebral mucormycosis with infiltration of the orbital cavity, or nasal sinuses with affection of oculomotor, abducent, trochlear and optic nerves with or without cavernous sinus thrombosis [42, 43].

Encephalitis is also reported with SARS-CoV-2 that is explained as direct invasion of the virus through the nasal cavity and backflow through the olfactory nerve to the brain. Yet evidence supporting this explanation is weak as analysis of cerebrospinal fluid of patients along plenty of cases failed to detect the viral RNA through reverse transcription polymerase chain reaction (PCR). Yet encephalitis with SARS-CoV-2 is suggested to be a result of direct immune reaction releasing cytokines, chemokines and inflammatory biomarkers within the central nervous system [8, 38].

Peripheral nervous system is also involved in acute cases. Many reports spoke about peripheral neuropathy and autoimmune reactions with autoantibodies against the peripheral nervous system causing Guillain–Barre spectrum syndromes few weeks after a negative PCR [44].

Many of the cases that recovered the acute phase of illness suffer a diverse natured symptom; such cases are termed long Covid. Neurological and psychiatric symptoms are the commonest in long Covid. Such symptoms range between fatigue, lack of concentration, inattention, memory lapses, generalized weakness, sleep disturbance, depressive or anxiety symptoms, post-traumatic stress, chronic headaches, autonomic dysfunctions. Psychiatric manifestations may reach up to delusions [45, 46].

Long Covid symptoms are much like the myalgic encephalomyelitis/chronic fatigue syndrome symptoms [47].

Autopsy of the brain of SARS-CoV-2 victims showed widespread of macrophages and inflammatory infiltrates as well as microglia within the brain and disruption of the blood brain barrier; such findings highlight the possibility of developing neurodegenerative conditions in the future as Parkinson’s and Alzheimer’s diseases [8, 48]

As for young children who catch SARS-CoV-2, the usual symptoms are mild and do not extend beyond the general viremia signs. Yet, few develop the rare multisystem-inflammatory syndrome in children (MIS-C) which is associated with excessive endothelial activation and this might be accompanied by neurological manifestations that range between mild symptoms as headache and anosmia, and can extend up to meningitis, seizures, encephalopathy, cerebellar ataxia, proximal myopathy and bulbar palsy. Such neurological manifestations are secondary to immune system overactivation with autoimmune reactions [49].

Unlike SARS-CoV-2, MPX virus has few reported neuropsychiatric manifestations, yet such statement might be misleading in the context that MPX cases are still in the range of thousands if compared to millions studied cases in SARS-CoV-2.

Reported neuropsychiatric manifestations of the endemic MPX are headache, myalgia, photophobia, pain and fatigue, seizures, encephalitis, anxiety, and depression that can mount up to suicide [50–52].

Fatigue, myalgia, and headache can be attributed to the viremia that is common with any viral infection [53]. As for psychiatric manifestations that ranges from anxiety up to major depression with suicidality it could be explained on basis of fear of stigma as most historical patients were hospitalized in quarantine hospitals [54].

Encephalitis was reported in cases along endemic regions of west and central Africa and also was previously reported in 2 pediatric cases both were diagnosed by MPX, and only one of them was subjected to cerebrospinal fluid analysis that showed antibodies (IgM) against the virus yet no evidence of the virus itself which could be explained on immunological basis rather than direct invasion of the brain by the virus [55, 56].

Along the current cross-border outbreak besides three deaths in Nigeria, two in Central African Republic, and one in Ghana, there are 2 reported deaths in Spain, one in Brazil and one in India. The two deaths in Spain are secondary to encephalitis and meningoencephalitis.

Despite SARS-CoV-2 manifestations in children are mild yet manifestations of MPX in children may be severe [56].

### Preventive measures and management

Being viruses, the general recommendation by different health authorities is just symptomatic treatment for symptoms as the use of analgesics and antipyretics to guard against constitutional symptoms as fever, headache and body aches is applied.

As for current National Institutes of Health (NIH) guidelines for SARS-CoV-2 it is divided into two phases. The first one is targeting the virus itself while in the phase of early infection and replication and the second one is targeting the dysregulated immune system [57].

Aggressiveness of treatment is also based on the illness status whether mild, moderate or severe as well as critically ill. For those who are not hospitalized, dexamethasone or any kind of systemic corticosteroids is not recommended.

As for those patients who are not hospitalized but are at risk of passing into severe form of SARS-CoV-2 infection as those who are immunocompromised or diabetics with uncontrolled diabetes, antiviral medications are to be administered as ritonavir-boosted nirmatrelvir or remdesivir and in case of unavailability then bebtelovimab or molnupiravir could be administered [57].

Hospitalized patients who do not require oxygen supplementation are managed as non-hospitalized but with a prophylactic dose of heparin. Meanwhile, hospitalized patients who are on oxygen support are supplied with dexamethasone besides remdesivir and full-dose heparin in case of elevated d-dimer and not pregnant. As for pregnant patients, prophylactic dose of heparin is recommended.

Intravenous tocilizumab is kept for hospitalized patients with rapid oxygen demands or systemic inflammation. Dexamethasone, remdesivir and intravenous tocilizumab are administered to critically ill patients on high-flow oxygen, non-invasive oxygen, or mechanical ventilation.

As for preventive measures directed against SARS-CoV-2, it is recommended to keep a distance not less than 2 m on dealing with a patient, and an infected patient should wear a face covering, with frequent hand washing of caregiver as well as the patient.

Vaccines are the most reliable preventive ways against SARS-CoV-2 infection. There are four main types of vaccines: whole virus vaccine, RNA or mRNA vaccine, non-replicating virus vector vaccine, and protein subunit vaccine [58].

MPX does not have a definite treatment yet. Brincidofovir and tecovirimat are under investigation for possibility of being effective based on previous partial success on sporadic cases in the United Kingdom. Such medications are still not recommended as a general treatment for all cases, but are left for those immunocompromised, or severely ill [59, 60].

Again, vaccines are the only way to guard against wide spread of MPX. Historically smallpox vaccine used to cause cross-immune protection against other viruses belonging to poxviridae family including MPX. The WHO is recommending to administer new forms of smallpox vaccine for those in medical field who deal with cases of MPX or as a prophylactic treatment within 4 days of contact with a case [61].

## Conclusion

Novel SARS-CoV-2 took the world by surprise when it emerged as an epidemic and within few months became a pandemic. SARS-CoV-2 has plenty of neuropsychiatric manifestations that play a great deal in its morbidities. As for MPX, it is an already known virus yet still surprising the unknown mode through which it crossed borders from its endemic areas in west and central Africa to other continents.

Mutations that have been already detected in MPX may have played a role in escaping its endemic region to other regions as well as suspicion of SARS-CoV-2 role in causing mass immunity reduction facilitating the spread of MPX as an opportunistic virus.

As SARS-CoV-2, monkeypox has neuropsychiatric challenges. The world health authorities still have a chance to control and manage MPX and prevent a potential pandemic through lessons learned from SARS-CoV-2.

## Limitations and future directions

The current review has some limitations. First, it is an initial narrative review as data regarding monkeypox manifestations are still minimal. Future in-depth reviews including systematic ones will for sure add to the current review.

Second, although presenting a link between two viruses one of them was never faced by the world and another that did not spread to such extent worldwide before although known since the middle of the twentieth century yet such presentation ought to be laboratory checked to confirm the role of lowered immunity caused by one virus in facilitating the spread of another.

Third, indirect role of improper distribution of corona virus vaccine along developing countries in monkeypox spread needs in-depth research and accordingly proper future guidelines on medical services and preventive medicine distribution ought to be implemented by the world health authorities.

## Data Availability

The corresponding author takes full responsibility for the data, has full access to all of the data, and has the right to publish any and all data separate and apart from any sponsor.

## Abbreviations

SARS-CoV-2: Severe acute respiratory syndrome corona virus 2
SARS-CoV: Severe acute respiratory syndrome corona virus
MERS-CoV: Middle east respiratory syndrome corona virus
MPX: Monkeypox virus
WHO: World Health Organization
ACE-2: Angiotensin converting enzyme 2
PCR: Polymerase chain reaction
IL: Interleukin
TNF-α: Tumor necrosis factor-alpha
MIS-C: Multisystem inflammatory syndrome in children
NIH: National Institutes of Health
